# A Case of Enlarged Intracranial Translucency in a Fetus with Blake's Pouch Cyst

**DOI:** 10.1155/2014/968089

**Published:** 2014-01-09

**Authors:** Ambra Iuculano, Maria Angelica Zoppi, Rosa Maria Ibba, Giovanni Monni

**Affiliations:** Department of Obstetrics and Gynecology, Prenatal and Preimplantation Genetic Diagnosis, Ospedale Microcitemico, Via Jenner, Cagliari, 09121 Sardinia, Italy

## Abstract

The intracranial translucency (IT) is a recently introduced marker of open spina bifida (OSB). In this study, we describe a case of a fetus affected by Blake's pouch cyst which showed alterations of BS/BSOB ratio at the first trimester screening.

## 1. Introduction

Intracranial translucency (IT) is an open spina bifida (OSB) marker at first trimester scan introduced by Chaoui et al. [1, 2]. IT represents the future 4th ventricle and its obliteration or reduction of its thickness is present in fetuses affected by OSB. This data is subject to ample discussion [3–5]. Recent studies show that the parameter, which best indicates the OSB risk, is the inversion of the thickness ratio between BS (brain stem) and brain stem to occipital bone distance (BSOB) BS/BSOB [6]. Numerous studies highlight the alteration of IT in several pathologies of the posterior fossa [6–9]. Nizard et al. [7] report four cases of fetuses with Dandy Walker syndrome, with posterior fossa changes starting the first trimester and demonstrating enlarged IT. Other recent publications confirm this observation [8–10]. Recently Lafouge et al. [11], reported enlarged IT in a fetus with Blake's pouch cyst (BPC). Garcia-Posada et al. reported 2 cases of BPC, and they described the Cisterna Magna (CM) which was enlarged [12].

We report a new case of BPC, in which the posterior fossa images of first trimester have been evaluated.

## 2. Case Report

**Figure 1:**
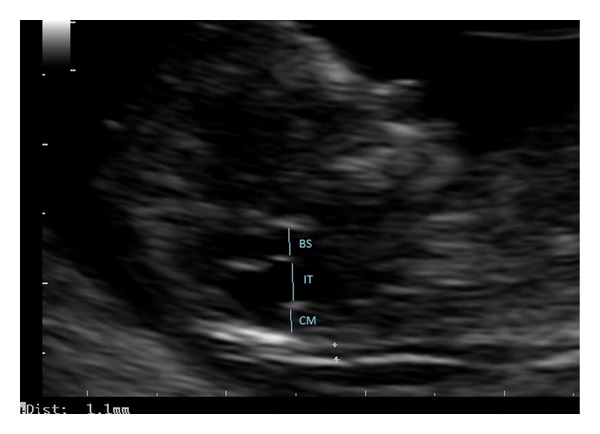
Fetal ultrasound image (sagittal view) at 11 + 4 weeks of gestation, showing an enlarged intracranial translucency (IT) in the posterior fossa, the brain stem (BS), and the cisterna magna (CM).

**Figure 2:**
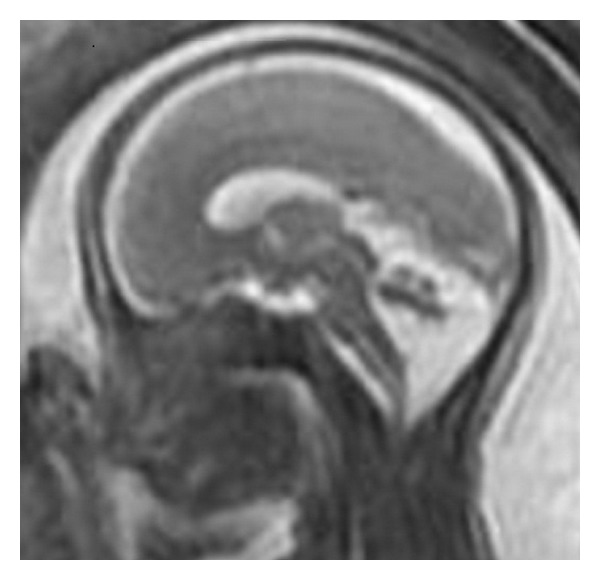
Second trimester fetal RMN (sagittal T2 weighted image), showing the posterior cranial fossa with the complete vermis. Persistent Blake pouch cyst was diagnosed.

A 29-year-old patient, G3 P1, underwent 1st trimester aneuploidy screening at 11 + 4 weeks of gestation (Figure 1). The crown-rump length was 52 mm, nuchal translucency was 1.1 mm, and the combined risk for trisomy was 21 < 1/10,000. At 21 weeks, the patient returned for suspected diagnosis of enlarged CM. At the ultrasound scan a connection between the CM and the 4th ventricle was evident and in the sagittal plane the cerebellar vermis was rotated. However, its biometry was normal [13]. The brainstem-vermis angle and brainstem-tentorium angle was determined by the method of Volpe et al. [14] and Ghi et al. [15]. Persistent BPC was diagnosed. Magnetic resonance was performed and the ultrasound diagnosis was confirmed (Figure 2). The pregnancy concluded uneventfully and the neonate showed normal neurological findings at birth. The neonatal transfontanellar ultrasound confirmed the prenatal diagnosis. At one month he has regular psychomotor development.

We reviewed retrospectively our first trimester screening ultrasound images and noted that the brainstem was thinner than expected. We measured the IT (3 mm), which was over the 95th percentile [1]. The BS diameter, BSOB, and the BS/BSOB ratio were evaluated. The ratio was 0.4 under the 5th percentile by Lachmann et al. [6]. The CM diameter (2.2 mm) was at the 5th percentile [12] and BS diameter (2.2 mm) was at the 95th percentile [6].

## 3. Discussion 

We report a case of a fetus affected by Blake's pouch cyst diagnosed prenatally and confirmed postnatally which presented an abnormal IT value as well as altered BS/BSOB ratio detected by first trimester ultrasound scan.

The Blake's pouch cyst is an embryological remnant, as an outpouch of the 4th ventricle in the posterior fossa, that persists beyond the 2nd trimester and may be present at birth. It is considered to be a benign entity, in most cases disappearing without sequela, while disturbances in the circulation of the cerebrospinal fluid can occur. Prenatal ultrasound diagnosis and neonatal followup is considered of importance.

Our retrospective case confirms the observation of Lafouge et al. showing that an alteration of IT is present in a case of fetus affected by Blake's pouch cyst. Besides, we have calculated the BS/BSOB ratio that was decreased; however, the CM and BS were in the limits of the normal range.

Garcia-Posada et al. [12] report two cases of Blake's pouch cysts that differ from the one already presented by Lafouge et al. [11] because they describe an enlarged CM rather than an enlarged IT.

Furthermore, they comment that the preferred indicator of BPC is an enlarged CM, having an efficacy similar to the one of the BS/BSOB ratio.

However, if the IT is not always obliterated and not easily identified and therefore is not the best indicator of OSB, then neither is the CM.

Also, Nizard et al.'s study [7] was performed in a period when the Blake's pouch cyst was considered as a Dandy Walker variant [16, 17]; therefore, it was important to identify it as soon as possible in order to take timely decisions on the managing of the pregnancy. However, after the BPC was reconsidered as a benign pathology [18, 19], the importance of these new first-trimester signs has changed. In fact, it is impossible to differentiate posterior fossa cystic diseases in the first trimester as benign (BPC, mega cysterna magna) or pathologic (Dandy Walker malformation).

According to our observation, it is important to perform the second trimester sonogram in order to establish a differential diagnosis. Above all, it is necessary to identify first trimester posterior fossa anomalies, because Dandy Walker malformations and sometimes BPC and mega CM are linked to different syndromes and/or chromosomal problems. Therefore, it is useful to examine the fetal anatomy, to find other pathological signs and, if necessary, to perform further diagnostic tests.

In conclusion, routine assessment of posterior fossa during first trimester screening is useful to be performed at the first trimester screening. The IT represents an important marker for posterior fossa pathologies; however, the evaluation of BS/BSOB is a better indicator for OSB as well as posterior fossa pathologies. It is suggested to wait until the second trimester so as to distinguish between benign and pathologic posterior fossa anomalies; yet, we need to obtain more details of its evaluation in the first trimester in order to validate this important data.
